# Dental adhesive microtensile bond strength following a biofilm-based *in vitro* aging model

**DOI:** 10.1590/1678-7757-2019-0737

**Published:** 2020-06-24

**Authors:** Aditi JAIN, Steve R. ARMSTRONG, Jeffrey A. BANAS, Fang QIAN, Rodrigo R. MAIA, Erica C. TEIXEIRA

**Affiliations:** 1 University of Iowa Department of Operative Dentistry Iowa CityIA USA University of Iowa, Department of Operative Dentistry, Iowa City, IA, USA.; 2 University of Iowa Iowa Institute for Oral Health Research Iowa CityIA USA University of Iowa, Iowa Institute for Oral Health Research, Iowa City, IA, USA.

**Keywords:** Degradation, Streptococcus mutans, Microtensile bond strength, Bonding, ISO standards

## Abstract

Laboratory tests are routinely used to test bonding properties of dental adhesives. Various aging methods that simulate the oral environment are used to complement these tests for assessment of adhesive bond durability. However, most of these methods challenge hydrolytic and mechanical stability of the adhesive- enamel/dentin interface, and not the biostability of dental adhesives.

**Objective:**

To compare resin-dentin microtensile bond strength (μTBS) after a 15-day *Streptococcus mutans* (SM) or *Streptococcus sobrinus* (SS) bacterial exposure to the 6-month water storage (WS) ISO 11405 type 3 test.

**Methodology:**

A total of 31 molars were flattened and their exposed dentin was restored with Optibond-FL adhesive system and Z-100 dental composite. Each restored molar was sectioned and trimmed into four dumbbell-shaped specimens, and randomly distributed based on the following aging conditions: A) 6 months of WS (n=31), B) 5.5 months of WS + 15 days of a SM-biofilm challenge (n=31), C) 15 days of a SM-biofilm challenge (n=31) and D) 15 days of a SS-biofilm challenge (n=31). μTBS were determined and the failure modes were classified using light microscopy.

**Results:**

Statistical analyses showed that each type of aging condition affected μTBS (p<0.0001). For Group A (49.7±15.5MPa), the mean μTBS was significantly greater than in Groups B (19.3±6.3MPa), C (19.9±5.9MPa) and D (23.6±7.9MPa). For Group D, the mean μTBS was also significantly greater than for Groups B and C, but no difference was observed between Groups B and C.

**Conclusion:**

A *Streptococcus mutans*- or *Streptococcus sobrinus*-based biofilm challenge for 15 days resulted in a significantly lower μTBS than did the ISO 11405 recommended 6 months of water storage. This type of biofilm-based aging model seems to be a practical method for testing biostability of resin-dentin bonding.

## Introduction

Although dental composite restorations demonstrate favorable immediate bonding properties, failures occur over clinical service due to secondary caries, marginal defects or staining, chipping, and fractures.^1^ The degradation of the resin-dentin interface is considered the weak link and it is cited as one of the main reasons for failure of composite restorations.^2^ These facts, as well as newer dental adhesives claiming improved bonding effectiveness with simplified application techniques, emphasize the relevance of testing the resin-dentin interface integrity before dental bonding agents are marketed.

Clinical trials provide an accurate and effective determination of the long-term bond effectiveness for new materials, and they constitute the highest standard for testing them.^3,4^ However, due to time and cost limitations, testing bond strength and conducting margin analysis in a laboratory are popular substitutes for clinical trials.^5^ The International Standards Organization (ISO) created the ‘ISO/TS 11405:2015 Dental Materials to standardize testing for these materials – testing of adhesion to tooth structure guideline which includes information about selecting substrates, storing and handling samples, the essential features of the various laboratory tests, including tests of microleakage, tensile bond strength, and marginal gaps.^6^ ISO 11405 (test type 3) recommends a water storage time of 6 months at 37ºC to “show durability of the adhesive bond.” Additional *in vitro* techniques for simulated aging including thermocycling and mechanical loading are commonly reported in the literature. *In vitro* bond strengths, when involving an aging method, have been correlated with the results of clinical trials.^3,4,7^ However, these techniques present disadvantages to simulated aging, which ultimately limits their use. Water storage is time-consuming, requiring a minimum of 6 months.^6,8^ Thermocycling methods lack a consensus regarding the ideal number of cycles or cycling protocol.^9^ Furthermore, these methods challenge mechanical and hydrolytic stability only, leaving enzymatic stability untested. Therefore, it would be desirable to adopt a more relevant method for simulated aging in a laboratory that can simultaneously speed the aging process for resin-dentin interfaces and help evaluate an adhesive long-term bonding properties.

In the oral cavity, bacteria can form biofilms on both soft and hard tissue, which includes a variety of restorative materials, such as ceramics, resin composites, and amalgams.^10^ Plaque bacteria and resin-based dental materials interact dynamically. Studies have shown that a greater amount of dental plaque develops on composites made of resin than on other kinds of restorative materials^11,12^ and a greater percentage of bacteria is viable for dental composites than for other restorative materials.^13^ Laboratory studies have also shown that incomplete polymerization or disintegration of resin-based composites can result in monomers leaching into the oral cavity, which can increase the virulence and promote growth of *Streptococcus mutans, Streptococcus sobrinus*, and *Lactobacillus*.^10,14-16^ In turn, studies have demonstrated the degradative effect of *S. mutans*-based biofilm on surface roughness of dental composites^11^ and integrity of resin-dentin interface.^17-20^These facts emphasize the relevance of testing for biostability when assessing bonding properties of dental adhesive. This study aims to determine whether the resin-dentin microtensile bond strength (μTBS) after a biofilm challenge using one of two cariogenic bacterial species, either *Streptococcus mutans* or *Streptococcus sobrinus* for 15 days was comparable or possibly even lower than that adhesive μTBS after an ISO 11405 type 3 test of 6 months of water storage.

Few *in vitro* studies have assessed how biofilm challenges affect the mechanical properties of resin-dentin interfaces.^17,19,20^ A biofilm model offers the possibility of enzymatic, acidic and hydrolytic degradation and thereby more effectively simulates *in vivo* conditions. The null hypothesis was that a biofilm challenge using *Streptococcus mutans* or *Streptococcus sobrinus* for 15 days would result in a dentin bond presenting approximately the same μTBS reduction as it would after water storage for 6 months.

## Methodology

### Overview

This study follows the guidelines for substrate selection and sample storage as recommended by ISO/TS 11405 standards.^6^ In total, 31 human molars with no caries or restorations were randomly selected from a group of extracted teeth available at the Iowa Institute for Oral Health Research, Iowa City, IA. Teeth were exempt from Institutional Review Board (IRB) because each extraction was performed for purely clinical reasons. Furthermore, the teeth could not be connected to the patient from which they were extracted.

### Specimen Preparation

Thirty-one extracted teeth were cleaned and stored in 0.5% Chloramine-T trihydrate bactericidal reagent until they were mounted in dental stone using a customized mold. The teeth were then trimmed to create a flat coronal dentin using a water-cooled diamond wheel (Whip Mix, Louisville, KY, USA) and the Computer Numeric Controlled (CNC) machine (University of Iowa, Iowa City, IA, USA). The exposed dentin surface was etched with 37% phosphoric acid (Kerr, Orange, CA, USA) for 15 seconds followed by application of Optibond FL primer and adhesive (Kerr, Orange, CA, USA) using the manufacturer’s instructions. The adhesive was light cured for 30 seconds (>18J/cm^2^). Three increments of Z-100 dental composite (3M ESPE, St Paul, MN, USA) were built and each 2mm increment was cured for 40 s (>55 J/cm^2^). Optilux 500 curing light (Demetron/Kerr, Danburry, CT, USA) was used in the study with an excitant irradiance of 1390 mW/cm^2^ as determined using MARC^TM^RC (Managing Accurate Resin Curing-Resin Calibrator, BlueLight Analytics, Halifax, Canada). A radiometer (Demetron/Kerr, Danbury, CT, USA) was used to evaluate the stability of energy output throughout the study. Each bonded assembly was then segmented perpendicular to the resin-dentin interface into four sticks using an Isomet 1000 sectioning machine (Buehler, Lake Bluff, IL, USA). Each of the 2 mm x 2 mm resin-dentin stick was further trimmed using the CNC machine into a dumbbell with cross-sectional area of 0.5 mm^2^, gauge length of 1 mm, and radius of curvature or ‘neck’ of 0.6 mm. Dumbbells were sterilized by storing them in 0.5% Chloramine-T disinfectant reagent (0.5% of chloramine-T trihydrate with autoclaved water) for 24 hours followed by rinsing five times with autoclaved water before being exposed to bacterial challenge, to avoid obvious damage of the test specimens, in lieu of autoclaving.^21^

### Simulated Aging

**Figure 1:**
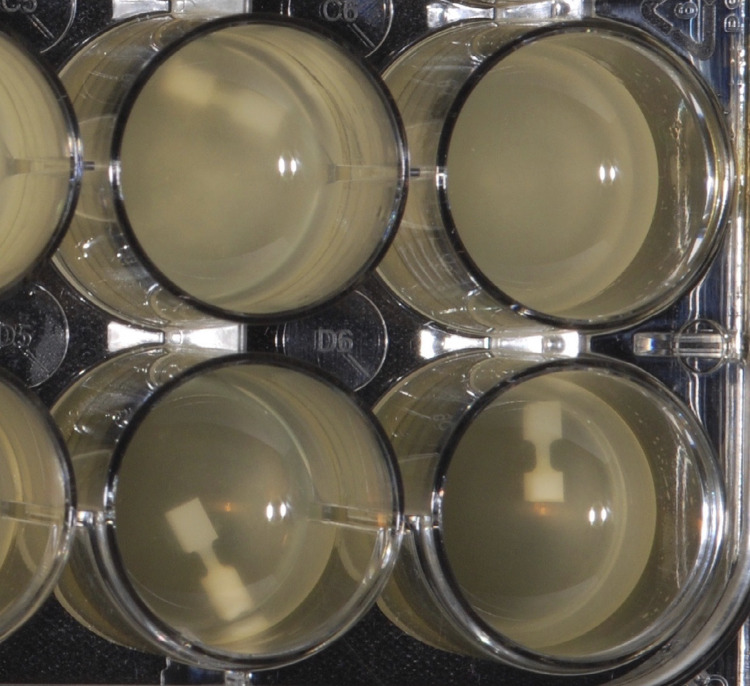
Dumbbells in bacterial aging medium

From each tooth, the 4 dumbbells were randomly placed in a 4-row by 6-column tissue culture plate (Costar 3526, Corning Inc., Corning, NY). For each of the four different tests for simulated aging, each well of a row of the plate contained 1 ml of the aging solution kept at 37 ^0^C. Storage conditions for the study were: A) 6 months in autoclaved water (n=31), B) 5.5 months in autoclaved water followed by exposure to 15 days of *Streptococcus mutans* (n=31), C) a 15-day *Streptococcus mutans*-based biofilm challenge (n=31), and D) a 15-day *Streptococcus sobrinus*-based biofilm challenge (n=31). The bacterial growth medium was changed daily, and the autoclaved water was changed, once every week. Frozen collection of *S. mutans* (UA159) and *S. sobrinus* (ATCC 33478) were revived for 24 hours on blood agar (Trypticase Soy Agar with 5% sheep’s blood) to generate the bacterial biofilms. A sterile Q-tip was used to inoculate the bacterial colonies into Brain Heart Infusion (BHI) broth from the agar plates. BHI broth consists of 6g brain heart (infusion from solids), 6g peptic digest of animal tissues, 5g sodium chloride, 3g dextrose, 14.5g pancreatic digest of gelatin and 2.5g disodium phosphate per liter of purified water.^19^ To promote the formation of a biofilm on the dumbbells within each well, BHI medium with 0.5% sucrose was used for the initial 24 hours^19^(Figure 1). Sucrose was not used afterwards, since it would have led to excessive biofilm biomass and extremely acidic pH. The biofilms were incubated aerobically with elevated (5%) CO_2_.

### Microtensile Bond Strength Testing

Immediately after the removal from the aging media, μTBS testing was performed at room temperature. For each test specimen, a non-gluing passive gripping device (Dircks Device, University of Iowa, Iowa City, IA, USA) held the specimen centrally in relation to its test axis. At a crosshead speed of 1 mm/min on a calibrated Zwick Materials Testing Machine (Zwick Gmbh & Co., Ulm, USA), μTBS testing was performed. For each specimen, two fractured segments were observed under a light microscope with a magnification of 50X. Based on these observations, the failure mode was classified either as: apparently adhesive, cohesive substrate failure in dentin or dental composite, or mixed when involving the adhesive interface and either dentin or dental composite.

### Statistical Analysis

When considering tooth dependency (four specimens from the same tooth), a simple random effect in Mixed Model ANOVA was performed to evaluate how each method of simulated aging affected the μTBS. Additionally, a Weibull regression model was used to determine association between μTBS and the types of storage media by Wald chi-square test.^8,22,23^ The significance for all tests was 0.05, and the SAS for Windows version 9.4 (SAS Institute Inc., Cary, NC, USA) was used to perform data analysis.

## Results

**Figure 2:**
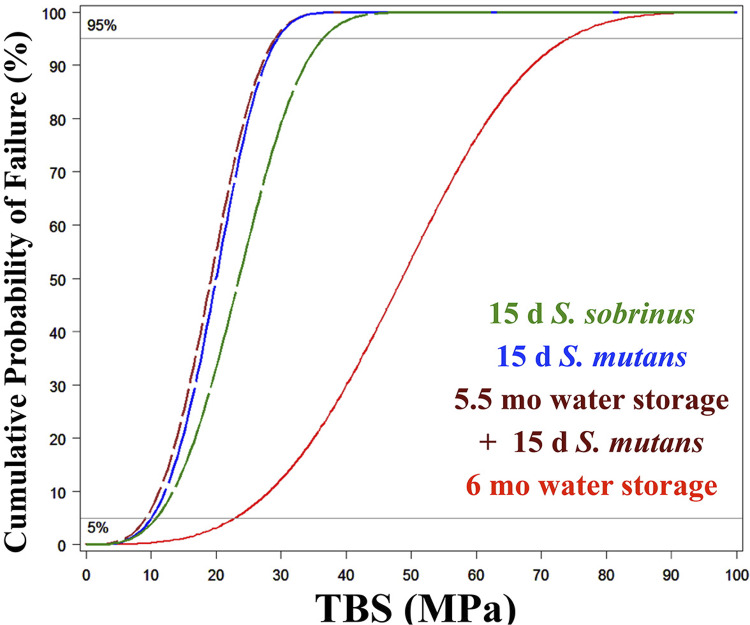
Weibull plot of probability of failure (%) against the microtensile bond strength (TBS) at failure for each of the storage conditions (d - days, mo – months, MPa – megapascal)

**Figure 3:**
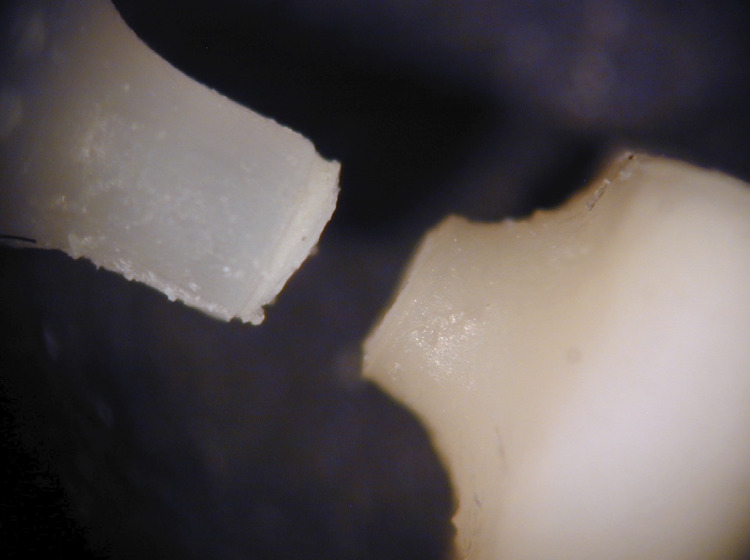
Apparent failure within dentin near the adhesive interface as seen under light microscopy (50X)

Mixed model ANOVA and Weibull regression model (Tables 1 and 2, respectively) showed that the method of simulated aging significantly (p<0.0001) affected the μTBS of dentin adhesive tested. Mean μTBS following 6 months of water storage was significantly higher than that following 5.5 months of water storage + 15 days of *Streptococcus mutans*, 15 days of *Streptococcus mutans* challenge or 15 days of *Streptococcus sobrinus* challenge (Table 1). Also, mean μTBS of specimens subjected to 15 days of *S. sobrinus* challenge was significantly greater than that following 5.5 months of water storage + 15 days of *S. mutans* challenge, or 15 days of *S. mutans* challenge, whereas no difference was found between the latter two groups (Figure 2). Weibull distribution was similar for all groups, as represented by the shape parameter. The scale parameter represented by η (eta) (63.2% probability of failure) is also shown in Table 2. Regarding the failure mode, most of specimens (74.2%, 83.9%, 80.6%) exposed to bacterial challenge (Groups B, C and D, respectively), had apparent cohesive failures within the dentin substrate, very close to the adhesive interface (Figure 3). In total, 55% of the specimens exposed to water challenge (Group A) had apparent cohesive failures within the dentin or dental composite substrate.

**Table 1:** Microtensile bond strengths associated with the four types of storage conditions analyzed using ANOVA

Groups (N=31)	Description of Aging Conditions	Mean (SD) Microtensile Bond Strength (Mpa)*
Group A	6 months Water storage	49.69 (15.53) ^A^
Group B	5.5 months Water storage + 15 days of *S. mutans* storage	19.26 (6.26) ^B^
Group C	15 days *S. mutans* storage	19.92 (5.86) ^B^
Group D	15 days *S. sobrinus* storage	23.58 (7.88) ^C^

*Column means with different letters indicate statistically significant differences

**Table 2:** Weibull parameters for the microtensile bond strength measurements associated with the four types of storage conditions

Storage Condition	N	Median* (stderr) (MPa)	95 % Confidence Interval	Scale (stderr)	95 % Confidence Interval	Shape (stderr)	95 % Confidence Interval	5% chance of Failure (stderr)	95 % Confidence Interval	95% chance of Failure (MPa) (stderr)	95 % Confidence Interval
6 mo WS	31	48.60 (2.86) A	43.30-54.54	54.01 (2.87)	48.68-59.94	3.47 (0.54)	2.55-4.71	22.93 (3.54)	16.94-31.05	22.93 (3.54)	65.35-84.08
5.5 mo WS + 15 d SM	31	19.22 (1.14) B	17.12-21.59	21.32 (1.13)	19.22-23.66	3.54 (0.52)	2.65-4.72	9.20 (1.36)	6.89-12.30	9.20 (1.36)	25.90-32.66
15 d SM	31	20.02 (1.11) B	17.95-22.32	22.05 (1.10)	19.99-24.31	3.79 (0.52)	2.90-4.96	10.08 (1.33)	7.78-13.05	10.08 (1.33)	26.54-32.65
15 d SS	31	23.54 (1.48) C	20.81-26.63	26.26 (1.48)	23.51-29.34	3.35 (0.47)	2.54-4.41	10.82 (1.64)	8.03-14.56	10.82 (1.64)	32.36-41.05

*Column medians with different letters indicate statistically significant differences (WS – water storage, mo – months, d – days, SS - Streptococcus sobrinus, SM - Streptococcus mutans, MPa – megapascal)

## Discussion

Based on the results, the null hypothesis was rejected. The lower μTBS results in this study following a biofilm challenge are consistent with the findings of other studies. Mutluay, et al.^19^ (2013) established that *S. mutans*-based biofilm storage for 14 days significantly reduced fatigue resistance and flexural strength of resin-dentin interfaces more than did water storage for 14 days. Carrera, et al.^20^ (2017) also found that 12 weeks of biofilm challenge resulted in larger reductions in the flexural strength of adhesive interfaces when compared to the control group without any biofilm challenge. Failure was within the demineralized enamel/dentin substrate close to the adhesive interface in both studies. This finding is consistent with that of this study. Moreover, groups presented similar Weibull shape parameters (moduli), which indicate that the defect variation among the groups was uniform.

The bacterial species, *S. mutans* and *S. sobrinus*, are acid-tolerant and among the strongest acidogens that may be found in dental plaque. These species metabolize dietary sugars into adhesive polysaccharides and organic acids.^24-27^*S. mutans* is more highly prevalent than *S. sobrinus,*^28,29^ so it was a natural choice for the biofilm model. *S. sobrinus* was added to this study to test it's effect on μTBS since *in vitro* studies have shown it as more acidogenic than *S. mutans*^28,29^ with perhaps higher caries potential.^24,30,31^ However, *S. sobrinus* biofilm caused less degradation of the resin-dentin interface, resulting in higher μTBS values than *S. mutans* biofilm. A preliminary experiment measuring the hourly change in pH observed a similar increase in acidity for both bacterial cultures, suggesting that acidogenicity does not explain why their biodegradation capabilities were different. However, such difference may be explained, by the lower probability of initial attachment of *S. sobrinus* and because that probability is increased when *S. mutans* is present with *S. sobrinus.*^24^

The rationale for including group B (5.5 months of WS + 15 days of SM) was to compare group B results and Group C (15 days of SM). If Group B μTBS was lower, then the combination of 5.5 months of WS + 15 days of SM-based biofilm challenge would be a more effective manner of *in vitro* aging than SM alone. However, as we did not find a difference between both groups likely signifies that most of the degradation presented in Group B (5.5 months of WS + 15 days of SM) was due to SM leading to low μTBS.

In past studies, researchers have investigated how a resin-dentin interface degrades when challenged with a multi-species biofilm.^20,32,33^ Although more clinically simulative, this kind of multi-species biofilm challenge is hard to control and standardize, since one of the bacterial species might dominate and outgrow the others. In this study, single species biofilms developed from *S. mutans-* and *S. sobrinus* were used. The strain of *S. mutans* selected for the study was UA159. This strain was chosen for it is well-studied and exhibits greater esterase activity on resin substrates, which are common in dental restorations, than other *S. mutans* strains.^18^ The strain of *S. sobrinus* selected for the study was ATCC 33478. This strain was used for it displays typical properties of the species. For the first 24 hours, a BHI medium was supplemented with sucrose to establish a biofilm. Sucrose was not used afterwards to avoid *S. mutans* to bind to and build up too much on the specimens for the remainder of the biofilm challenge. If active gripping with glue was used to test for bond strength, a larger biomass would have created difficulties, but since we used a mechanically passive gripping device without glue, it did not affect our study.

The μTBS test was used instead of the traditional macro-shear test due to improved stress distribution at the true resin-dentin interface and to achieve accelerated degradation at short diffusional distance and relatively larger adhesive dentin margin exposure.^7^ Despite being technically demanding, cylindrical dumbbell-shaped specimens were used instead of rectangular resin-dentin beams due to more uniform stress distribution at the dentin-resin interface under tensile load, thereby providing more reliable results.^7,34^ For data analysis of the results, both ANOVA procedures and a Weibull regression model were used. The Weibull regression analysis is highly recommended, since it can account for variations in μTBS results and for clusters of samples that occur when four dumbbells obtained from a single tooth are used.^8,22,35^ Most research studies avoid the test because of the large sample size it requires.^22^ In this study, a sample size one-third larger would have been adequate if we were to use only ANOVA for data analysis.

Future work may address some limitations of this study. Fractography and tracing the bacterial infiltration pathway using Scanning Electron Microscopy or Confocal Laser Scanning Microscopy would be useful for understanding biodegradation. Our study did not include an uninoculated BHI control. However, a significant difference was found between μTBS values of groups exposed to *S. mutans* and to *S. sobrinus,* therefore indicating probable degradation due to the bacteria used, and not the BHI media. Furthermore, a study measuring the quantity of Bis-GMA-derived degradative product bishydroxy-propoxy-phenyl-propane (Bis-HPPP) did not find a significant difference in degradation of composite (Z-250) and total etch adhesive (Scothcbond Multipurpose) following 14 and 30 days of BHI media when compared to baseline levels at 2 and 4 days.^18^

In the future, a different specimen design can also be tested to complement the biofilm challenge, such as the recently developed mini-interfacial fracture toughness test.^36^ Although this study found that cariogenic bacteria, *S. mutans* and *S. sobrinus*, result in biodegradation, it would be helpful to investigate whether non-cariogenic, acid-producing bacteria, including Streptococcus *sanguinis, Streptococcus gordonii*, or *Streptococcus mitis*—three species often found in dental plaque—also result in biodegradation. It is also important to understand that different degradation mechanisms occur at distinct parts of the restorations and enamel or dentin tooth substrates. Future studies could compare exposed restored teeth with and without enamel margins as well as individual test specimens; however, we chose to study the resin-dentin bond as this is a common restorative cavosurface margin and site of margin degradation to include recurrent caries. The resin-dentin interface is the weak link of adhesive dentistry due to the dentin substrate nature and the *in vivo* degradative mechanisms.

In summary, biofilm challenge for just 15 days produced significantly greater degradation and resulted in much lower μTBS values than did water storage for 6 months. This suggests that a biofilm challenge used to evaluate the hydrolytic and biostability of dental adhesives has a clear purpose while testing mechanical properties of bonding agents in the laboratory. However, the extent to which this model decreases the resin-dentin bond strength when compared to non-aged specimens cannot be determined in this study. The bacterial challenge can be helpful to assess how newer antibacterial resin monomers bond to dentin substrate.^37^ The method of simulated aging in this study has been deliberately kept simple and it is easily to reproduce.

The biofilm-based model seems to be a promising *in vitro* method for simulated aging. However, this area needs further refinement and exploration into how well it complements or can replace other *in vitro* aging models dedicated to testing μTBS properties of dental adhesives.

## Conclusion

Within the limitations of this *in vitro* study, a *Streptococcus mutans-* or *Streptococcus sobrinus*-based biofilm challenge for 15 days resulted in a significantly lower μTBS than the ISO 11405 recommended 6 months of water storage. This type of biofilm-based aging model seems to be a practical method for testing biostability of resin-dentin bonding.
